# Exercise-Induced Immunodepression in Endurance Athletes and Nutritional Intervention with Carbohydrate, Protein and Fat—What Is Possible, What Is Not?

**DOI:** 10.3390/nu4091187

**Published:** 2012-09-04

**Authors:** Wolfgang Gunzer, Manuela Konrad, Elisabeth Pail

**Affiliations:** Department of Dietetics and Nutrition, University of Applied Sciences FH JOANNEUM, Kaiser-Franz-Josef-Strasse 24, Bad Gleichenberg 8344, Austria; Email: wolfgang.gunzer.dio09@fh-joanneum.at (W.G.); elisabeth.pail@fh-joanneum.at (E.P.)

**Keywords:** exercise-induced immunodepression, macronutrients, URTI, immune function

## Abstract

Heavily exercising endurance athletes experience extreme physiologic stress, which is associated with temporary immunodepression and higher risk of infection, particularly upper respiratory tract infections (URTI). The aim of this review is to provide a critical up-to-date review of existing evidence on the immunomodulatory potential of selected macronutrients and to evaluate their efficacy. The results of 66 placebo-controlled and/or crossover trials were compared and analysed. Among macronutrients, the most effective approach to maintain immune function in athletes is to consume ≥6% carbohydrate during prolonged exercise. Because inadequate nutrition affects almost all aspects of the immune system, a well-balanced diet is also important. Evidence of beneficial effects from other macronutrients is scarce and results are often inconsistent. Using a single nutrient may not be as effective as a mixture of several nutritional supplements. Due to limited research evidence, with the exception of carbohydrate, no explicit recommendations to reduce post-exercise URTI symptoms with single macronutrients can be derived.

## 1. Introduction

The human immune system and its response to any specific stimulus is extremely complex and comprises a variety of physical elements, cell types, hormones and interactive modulators. These responses are precisely coordinated to protect the body’s tissues against pathogenic agents. Multiple factors influence the athlete’s resistance to illness, and the immune system can become functionally depressed. Examples of such factors include genetically predisposed immune competency, inadequate nutrition, physical, psychological and environmental stresses and alterations in normal sleep schedule [1].

Heavy training schedules or endurance competitions, such as marathons or long-distance cycling, are forms of extreme physical stress and lead to immunodepression in athletes, which is associated with increased susceptibility to infection, especially upper respiratory tract infections (URTI) [2,3]. Daily training regimens and competition performance may be disrupted, which is undesirable. Athletes are therefore interested in nutritional strategies in order to maintain immunocompetence and to avoid illness [4]. This review summarizes and evaluates the influence of poor dietary practices, nutrition state and the potential of macronutrients (carbohydrates, proteins and fats) working as a countermeasure to exercise-induced immunodepression in endurance athletes. Only nutritional intervention studies with the purpose of minimising post-exercise immunodepression in endurance exercise (running, cycling, rowing) with macronutrients were included. Trials comprising resistance exercise protocols or examining immunomodulation with anti-oxidants or dietary immunostimulants were excluded.

### 1.1. Endurance Exercise and Upper Respiratory Tract Infections

Several key studies investigating the incidence of URTI after prolonged endurance events were done during the 1980s and 1990s [5,6,7]. For example Peters and Bateman [5] studied the incidence of URTI following a marathon-type endurance event (distance of 56 km) in 150 randomly selected participants and compared them to 124 age-matched controls. During the 2-week post-race period 33.3% of the runners reported symptoms of URTI, compared with 15.3% in the control group. In addition, it was revealed that a high training distance per week (>65 km) could lead to more URTI symptoms than a lower weekly training distance/load. These initial findings were confirmed by a number of investigators [6,7,8,9,10,11], but not by all [12,13]. Even though exercise-induced immunosuppression is typically mild and transient [14], it has been of particular interest in the field of exercise immunology during the last two decades, because acute respiratory infections, sore throats and flu like symptoms may interfere with training and lead to a poor endurance performance in elite athletes [15,16,17].

The relationship between exercise intensity/volume and susceptibility to URTI has been modeled in the form of a “J” curve [18]. This model suggests that moderate exercise may lower the risk for URTI compared to sedentary individuals—it appears to be beneficial to a certain point [15]. On the other hand, high-intensity exercise and periods of strenuous exercise may raise the risk for URTI [18]. Although based on epidemiological data from observing or self-reporting of symptoms of URTI this model has been widely accepted by athletes, trainers and scientists [19,20]. However, to date there is still a lack of evidence of a direct link between heavy exercise and URTI in scientific literature or the results are inconclusive [15,16,20,21]. For example Moreira *et al*. [20] proposed a three-dimensional model of the J-shaped curve and hypothesized that a relation between exercise load and URTI would be expected to be more common in less fit athletes than in elite level athletes (high fitness level). In addition, three hypotheses concerning allergy, inflammation or infection as main causes for post-exercise URTI symptoms were discussed, but strong evidence is still lacking [16,22]. 

Since the underlying mechanisms are still unclear [23] it should be kept in mind that several other factors could also be partly responsible for the higher incidence of URTI experienced by athletes, such as environmental factors (e.g., heat), increased exposure to pathogens or as discussed later, poor nutritional status [19,24,25]. Nevertheless, there is documented depression of immune function—more precisely suppression of some immune variables—following heavy exertion lasting between three and 72 h [4,19,21]. During this time of impaired defense—referred to as the “*Open Window*”—pathogen resistance is lowered, thus increasing infection risk [4,19]. 

### 1.2. Effects of Heavy Exercise on Cellular Immune Function

Numerous studies have shown that exercise has either a positive or a negative effect on immunity. These effects depend on the nature, intensity and duration of exercise, as well as subject fitness and age and therefore outcomes are highly variable [17,26,27]. For example in young boys and girls (12 years old), changes in the immune function are smaller and recover more rapidly after strenuous cycling compared to adolescents (14 years old) [28]. In general post-exercise immune function impairment is highest when the exercise is continuous, prolonged (>1.5 h), of moderate to high intensity (50%–77% maximum O_2_ uptake (VO_2max_)), and performed without food intake [16].

#### Effects of Acute and Chronic Exercise on Immune Function

An acute bout of heavy exercise induces immune system responses, which are similar to those induced by infection [3]. An increase in circulating neutrophils, monocytes and natural killer (NK) cells [27,29], a catecholamine-mediated lymphocytosis [30] and a higher plasma concentration of several hormones (e.g., epinephrine, cortisol, growth hormone and prolactin) [3] can be observed. Furthermore an enhanced release of anti-inflammatory (e.g., IL-10, IL-1ra) and pro-inflammatory cytokines (e.g., TNF-α, IL-6, IL-1β, IL-8) [27] and acute phase proteins such as C-reactive protein (CRP) is induced [3]. The expression of toll-like receptors, proteins for recognizing pathogens, is reduced [31]. 

Immediately post-exercise or during early recovery the changes in leukocyte counts begin to return to resting levels [27], NK cell number and activity fall below pre-exercise levels [29], the lymphocytosis turns into a cortisol-induced lymphocytopenia before returning to resting values [30], and the neutrophil:lymphocyte ratio increases, which is an accepted indicator of exercise stress [32]. 

T-cell function and production decreases due to high stress hormone levels and exercise-induced alterations in the pro/anti-inflammatory cytokine balance [15,16], the oxidative burst (killing capacity) of phagocytic neutrophils is reduced for several hours [3], and plasma glutamine concentration may be decreased by about 20% [1]. Serum immunoglobulin (Ig) concentration remains unaffected or slightly increases, but there is a decline in salivary IgA (s-IgA) both in concentration and secretion rate [30]. These changes of immunity cell populations and functions during early recovery may lead to a higher infection risk and the above-named “*Open Window*” [3].

Periods of intensified training lasting for one week or more, frequently observed over the course of a competitive season or in underperformance syndrome, may result in chronically impaired immune function and increased infection risk [25,33,34]. Chronic effects of heavy exercise not only include a higher risk to URTI but also lowered numbers of leukocytes at rest compared to sedentary people, decreased neutrophil function, serum and salivary Ig concentration, NK cell number and possibly cytotoxic activity [16,35,36,37]. Several causes for impaired immune cell function due to repeated bouts of strenuous exercise are discussed [3]:

(a) Consequent elevated levels of stress hormones, particularly cortisol;
(b) Insufficient time between the bouts for immune system to recover fully;
(c) Plasma glutamine levels may become chronically depressed.

For example, Ronsen *et al*. [38] showed that a recovery time of 3 h between two bouts of strenuous endurance exercise results in higher levels of stress hormones and augmented immune cell dysfunction compared with 6 h of recovery between exercise bouts. Similar findings were presented by Degerstrom & Osterud [39] with a 4 h-rest interval between two consecutive bouts.

### 1.3. Influence of Nutrition State on Pre-Exercise Immune Function

**Figure 1:**
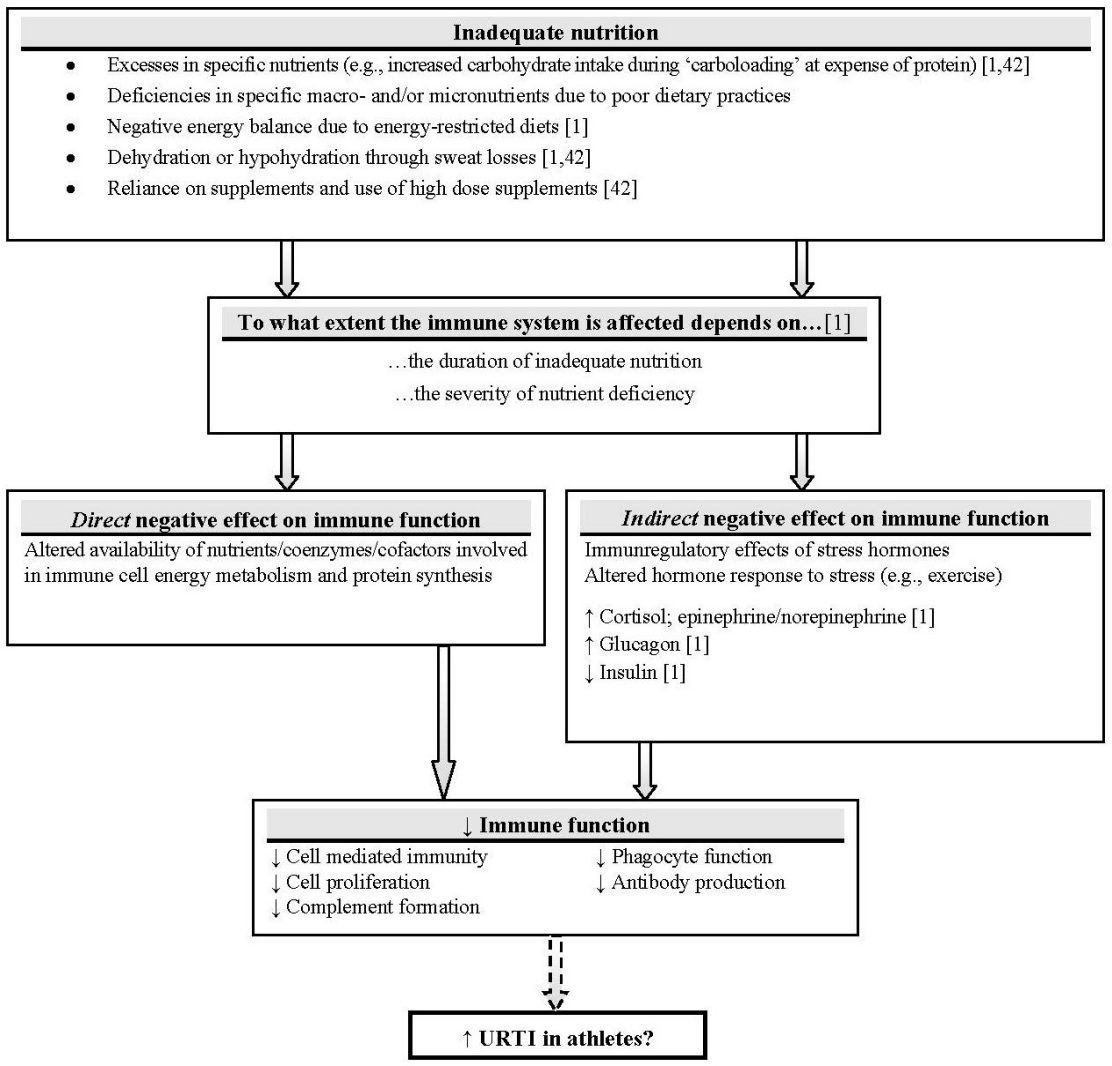
Nutrient availability and immune function: direct and indirect mechanisms. It can be inferred that a poor nutrition state may exacerbate cellular immune responses to heavy exercise and further impair immune function [47]. Adapted with permission from Walsh [32] (Solid arrows: research evidence mostly supports link; dashed arrow: limited research evidence to support link in athletes; ↑: increase; ↓: decrease).

Scientific research has long shown that inadequate nutrition may contribute to impaired immunity and makes the individual more susceptible to infection (Figure 1) [32,40]. Energy-restricted diets are common in sports, where low body fat is desired, such as running and cycling [41], and could be accompanied by macro- and micronutrient deficiencies [2]. Excesses in specific nutrients, such as carbohydrates at expense of protein, training in a dehydrated state and excessive use of nutritional supplements may also lead to direct and indirect negative effects on the immune function in athletes and may be partly responsible for higher infection risk [1,32,42]. Maintaining the normal function of immune cells requires an adequate amount of water, glucose, proteins and electrolytes [43]. As a logical consequence, meeting nutritional demands helps to maintain an effective immune system [42].

## 2. Nutritional Modulation of Exercise-Induced Immunodepression

Despite a large number of publications on possible immunomodulatory effects of selected macronutrients on exercise-induced hormonal and immune responses, the variety of employed methods, heterogeneity in the population sample (age, gender, fitness level), the effect of different exercise protocols (type, mode, duration and intensity) and the type, amount and timing (pre- or post-exercise, during exercise) of ingested nutrient make the comparative analysis difficult. Recent examinations found that variance in several exercise-induced changes of immunity e.g., cytokine response depends on exercise intensity [44,45,46]. To measure immunomodulation in human nutrition intervention studies Albers *et al*. [25] emphasized, that no single immunological marker allows conclusions to be made about efficacy. The best approach is to combine immunological markers with HIGH suitability (e.g., s-IgA) with MEDIUM suitable markers (e.g., NKCA, oxidative burst of phagocytes, lymphocyte proliferation and cytokine milieu).

### 2.1. Carbohydrate, Exercise and Immune Function

It is clear that an adequate amount of carbohydrate (CHO) availability is a key factor for maintenance of heavy training schedules and successful athletic performance [32,48,49]. As mentioned above maintaining the normal function of immune cells requires an adequate amount of glucose besides water, proteins and electrolytes [43]. Glucose is an important fuel substrate for lymphocytes, neutrophils and macrophages, because metabolic rates of immune cells are extremely high [1]. High levels of stress hormones such as cortisol and catecholamines (epinephrine, norepinephrine) not only occur during high intensity exercise but also depend on glucose availability [1]. A low level of blood glucose concentration during prolonged exertion results in higher levels of cortisol, epinephrine and growth hormone [26,50]. The immunosuppressive effects of acute and chronic stress and high levels of stress hormones are well established [4]. Thus, the underlying rationale is that adequate CHO availability and stable blood glucose concentration may limit stress hormone responses [1,14], provide glucose as energy substrate for immune cells [32] and help to maintain immunity [42].

#### 2.1.1. Availability of Dietary Carbohydrate

Several trials investigated the influence of pre-exercise carbohydrate fuel state on hormonal and/or immune response to endurance exercise [51,52,53,54,55,56]. In most cases participants performed a glycogen-depleting exercise (1 h cycling)—except in one study [55]—and were then set on a high (70%–77% dietary intake from CHO/8.0 g CHO/kg bodyweight (BW) per day (/day)) or low (7%–11% dietary intake from CHO/0.5 g CHO/kg BW/day) CHO diet for two to three days [51,52,53,54,55]. Costa *et al*. [56] allocated their subjects into a *self-selected* or *high* (12.0 g CHO/kg BW/day) CHO diet group for a 6-day period. After completing the diet, subjects had to perform a single bout of strenuous exercise—either 1 h of cycling ergometry at 70%–75% VO_2max_ [51,52] or at 60% Wmax followed by a time trial [53,54] or downhill running [55]. In the study of Costa *et al*. [56] participants had to run 1 h/day for six days in addition to their normal training regimens to create a cycle of overload training.

Depending on tested immunological markers, it was found that exercising on a high-CHO diet compared to a low-CHO diet leads to an increased [51,53] or stable [56] blood glucose level. Plasma cortisol levels may be decreased [51,53], the post-exercise glutamine level may rise [54] or stays unaffected [52]. Different effects on immune cell counts have been observed: lower numbers of neutrophils [53,55], an attenuated post-exercise leukocytosis [55], but also unaffected leukocyte counts [52], and unaffected post-exercise lymphocytopenia [51]. A high-CHO diet during times of intensified training for six days may have a favorable effect on mucosal immunity [56].

Training on low levels of CHO availability may raise the magnitude of exercise-induced immune alterations, such as higher plasma and salivary cortisol levels [52,56], decreased glutamine levels [52], higher number of circulating immune cells [51,52] and an enhanced cytokine response [54] (Table 1).

**Table 1:** Effects of pre-exercise high- *vs*. low-CHO diet on hormonal & immune response to endurance exercise (↑: increase; ↓: decrease; ↔: no effect; CHO: carbohydrate; BW: bodyweight; /day: per day).

Hormonal/Immune Response	High-CHO Diet (70%–77% Dietary Intake from CHO/8.0–12.0 g CHO/kg BW/day)	Low-CHO Diet/Self Selected (7%–11% Dietary Intake from CHO/0.5 g CHO/kg BW/day)
Glucose response	↑ Glucose response [51,53] ↔ [56]	↓ Low blood glucose level [56]
Glutamine level	↑ Glutamine level [51,57] ↔ [52]	↓ Glutamine level [52]
Cortisol response	↓ Plasma cortisol [51,53,58]	↑ Plasma or salivary cortisol [52,56]
Leukocyte & lymphocyte cell counts	↔ Circulating leukocytes [52]	↑ Numbers of neutrophils [52], leukocytes, lymphocytes [51]	
	↓ Numbers of neutrophils [53,55]Trend to attenuate post-exercise leukocytosis [55]		
		↑ Neutrophil:lymphocyte ratio [52,54]	
	↔ Post-exercise lymphocytopenia [51]		
Mucosal immunity	↑ Post-exercise s-IgA concentration than pre-exercise [56]	
Cytokine response		↑ IL-6, IL-10, IL-1ra [54]

The influence on some immune variables of a CHO containing meal with different glycemic indices (GI) and glycemic loads (GL) ingested 2–3 h before endurance exercise was also tested [59,60,61]. It was found that a pre-exercise meal with a high CHO amount (65% of energy intake) may attenuate exercise-induced cytokine response, and influences leukocyte trafficking [59]. The influence of pre-exercise meals consisting of low GI foods on exercise-induced cortisol and cytokine response compared to high GI meals remains still unclear because results are inconsistent [60,61].

Although limited evidence exists, it should be highlighted that exercising in a carbohydrate-depleted state, results in higher levels of circulating stress hormones [44], greater perturbations of immune cell subsets [43] and an impaired immune function [51]. Keeping the muscle and liver glycogen stores full is therefore a crucial factor [62].

#### 2.1.2. Carbohydrate Ingestion during Exercise and Immune Function

It is well established that CHO ingestion during high-intensity exercise improves athletic performance [48] and is widely practiced by athletes. Thus, effects of acute CHO ingestion on exercise-induced changes in immune function were extensively researched during the last 15 years. This section summarizes selected results from 29 placebo-controlled and/or crossover studies addressing this topic in which three [63,64,65] referred to the same subjects and exercise mode (Table 2).

A significant higher post-exercise blood glucose level in CHO supplemented groups (SUP) relative to controls (PLA) was shown in all presented studies. Due to the maintained blood glucose level, the majority of trials revealed an attenuated cortisol level, except three studies, where post-exercise cortisol levels in SUP did not differ from those in PLA [66,67,68].

Referring to Table 2, consuming a beverage delivering at least 6% CHO (1 L/h) during a minimum 1 h lasting endurance exercise of high intensity may help to attenuate exercise-induced increases of total leukocyte count and/or leukocyte subsets such as monocytes and neutrophils. Some researchers reported a lower post-exercise lymphocytosis [65,69,70,71,72,73] and a trend to attenuate lymphocytopenia during early recovery [65,74], but these findings were not confirmed by others [67,68,75,76,77,78].

Although NK cells are part of the innate immune system [79], only few attempts were made to evaluate effects of CHO ingestion during exercise on NK cells and function—with inconclusive outcomes. No significant difference between SUP and PLA in NK cell counts was shown by several investigators [67,74,76,77,78]. Contrary to this, Nieman *et al*. [65] reported a significant lower number of NK cells, which was confirmed by Timmons *et al*. [71]. In one study, cytotoxic activity of NK cells was reduced due to CHO supplementation [69].

As shown in Table 2 the effect of CHO ingestion on exercise-induced cytokine responses was investigated in 15 of the presented studies with contradictory results. It appears that acute CHO ingestion attenuates the cytokine response to prolonged exercise on particular cytokines, such as IL-6, IL-10 and IL-1ra but not on IL-8 and TNF-α. Mucosal immunity, particular saliva flow rate and s-IgA, was measured by only three investigators with no differences between SUP and PLA [68,70,80] but a higher saliva flow rate and lower s-IgA concentration in SUP compared to fluid restriction during exercise [81].

**Table 2:** Effects of CHO supplementation during exercise on selected immune variables relative to control. (↑: significant increase; ↓: significant decrease; ↔: no difference; -: not tested/not accessible; post: post-exercise; Wmax: maximal power; TT: time trial; PLA: control group; Ref.: Reference).

Ref.	Mode	Intensity	CHO dose	Leukocytes	Lymphocytes	Neutrophils	NK Cells	Cytokines	Mucosal Immunity	Cortisol	Blood Glucose	
[63]	2.5 h running	77% VO_2max_	6%	-	-	-	-	↓ IL-6 post, post 1.5 h	-	↓ post	↑ post	
								↓ IL-1ra post 1.5 h				
[64]	2.5 h running	77% VO_2max_	6% every 15 min	↓ (monocytes) post	↓ post	↓ post	-	-	-	↓ post	↑ post	
					↑ post 3 h	↓ post 1.5 h						
[65]	2.5 h running	77% VO_2max_	6% every 15 min				↓ post	-	-	↓ post	↑ post	
							↔ NKCA					
[82]	2.5 h running or cycling	75% VO_2max_	6% every 15 min	↓ (monocytes) post	-	↓ post	-	-	-	↓ post	↑ post	
[69]	2.5 h running or cycling	75% VO_2max_	6%	-	↓ post-exercise lymphocytosis	-	↓ NKCA	-	-	↓ post (cycling & running)	↑ post	
[68]	2 h cycling	60% VO_2max_	6%	-	-	-	-	-	↓ s-IgA concent-ration during exercise	-	↑ post	
[66]	2 h rowing	-	-	↓ (monocytes) post	-	↓ post	-	↓ IL-1ra post	-	↔	↑ post	
								↔ IL-6 post				
								↔ IL-8 post				
								↔ TNF-α				
[83]	1 h cycling and running	At individual’s lactate threshold	6.4% 12 mL/kg BW	-	-	-	-	↓ IL-6 post in cycling & running	-	-	↑ post	
[50]	Marathon run	-	6%	-	-	-	-	↓ IL-10 post	-	↓ post	↑ post	
								↓ IL-1ra post				
								↔ IL-6 post				
								↑ IL-8 post				
[84]	6 × 20 min cycling	90% of individual’s lactate threshold	1 g/kg BW/h (10%)	-	-	-	-	↓ cytokine response post	-	↓ post	↑ post	
[85]	6 × 15 min intermittent running	-	Every 15 min	-	-	↓ post 30 min	-	↓ IL-6 post 30 min	-	↓ post 30 min	↑ post	
								↔ TNF-α post				
[70]	3 h run	70% VO_2max_	6% every 15 min	↓ post	↓ post	-	-	↓ IL-6 post	↔	↓ post	↑ post	
								↓ IL-10 post				
								↓ IL-1ra post				
								↔ IL-8 post				
[86]	2 h cycling	-	6.4%	-	-	-	-	↓ IL-6 post	-	-	↑ post	
								↓ muscle derived IL-6 post				
[87]	2 h cycling	75% VO_2max_	6.4%	-	-	↓ post	-	-	-	↓ post	↑ post	
						↓ post 1 h						
[75]	2.5 h cycling	85% VO_2max_	6%	↓ post	↔	↓ post	-	-	-	↓ post	↑ post	
				↓ post 1 h		↓ post 1 h				↓ post 1 h		
[76]	Marathon run	-	6%	↓ (monocytes) post	↔ on post lymphocytopenia	↓ post	↔	↔ cytokine response post	-	↓ post	↑ post	
[77]	2 × 1 h cycling	75%–80% VO_2max_	60 g/h	-	↔	-	↔	-	-	-	↑ post	
[71]	1 h cycling	70% VO_2max_	6%	-	↓ post	↓ post	↓ post	↔ IL-6 post	-	-.	↑ post	
						↓ post 1 h		↔ TNF-α post				
[88]	2.5 h cycling	60% Wmax	6%	-	-	-	-	↓ IL-6 post	-	↓ post	↑ post	
								↓ IL-10 post				
								↓ IL-1ra post				
								↔ IL-8 post				
								↔ muscle IL-6, IL-8, TNF-α post				
[80]	2 × 1.5 h cycling	60% VO_2max_	10%	-	-	-	-	-	↔	↓ post	↑ post	
[72]	2.5 h cycling	65% VO_2max_	6.4%12.8%	↓ post in 6.4% + 12.8%	↓ post in 6.4% + 12.8% (T-cell subpopulations)	↓ post in 6.4% + 12.8%	-	-	-	↓ post in 6.4% + 12.8%	↑ post in 6.4% + 12.8%	
				↓ post 2 h in 6.4% + 12.8%		↓ post 2 h in 6.4% + 12.8%						
										↓ post 2 h in 6.4% + 12.8%		
				↔ between 6.4% + 12.8%						↔ between 6.4% + 12.8%		
						↔ between 6.4% + 12.8%						
[67]	1.5 h running on two consecutive days	70%–80% VO_2max_	6.4%	↓ total count (D1 + D2)	ND (D1 + D2) but ↓ T-cell count post (D1 + D2)	↓ post (D1 + D2)	↔	-	-	↔ (D1 + D2)	↑ post (D1 + D2)	
				↓ (monocytes) post (D1)								
						↓ post 1 h (D1 + D2)						
	DAY1 (D1)			↓ (monocytes) post 1 h (D1 + D2)								
	DAY2 (D2)											
[74]	4 h cycling	70% of individual anaerobic threshold	6%12%	↓ post in 6% + 12%	↔ but trend to attenuate lymphocytopenia in 6% + 12% post 1 h	↓ post in 6% + 12%	↔	↓ IL-6 post in 6% + 12%	-	↓ post in 6% + 12%	↑ post in 6% + 12%	
								↓ IL-6 post 1 h in 6% + 12%		↓ post 1 h in 6% + 12%		
				↓ post 1 h in 6% + 12%								
				↔ between 6% + 12%		↓ post 1 h in 6% + 12%						
						↔ between 6% + 12%						
								↔ between 6% + 12%		↔ between 6% + 12%		
[78]	2 h cycling	64% Wmax	6% every 15 min	↓ (monocytes) post	↔	↓ post	↔	-	-	↓ post	↑ post	
[89]	Duathlon (5 km run—20 km cycling—2.5 km run)	-	6% malto-dextrin	-	-	-	-	-	-	↓ post	↑ post	
[73]	2 h cycling	65% VO_2max_	6% CHO	↓ post in experimental conditions with CHO	↓ post in experimental conditions with CHO	↓ post in experimental conditions with CHO	-	-	-	↓ post in CHO/PLA condition	↑ post in experimental conditions with CHO	
			6 mg/kg BW caff eine (CAF)	↓ post 1 h in experimental conditions with CHO		↓ post 1 h in experimental conditions with CHO						
										↔ in CHO/CAF		
[68]	1.5 h cycling followed by 16 km TT	-	0,24 g/kg BW CHO gel every 15 min	↓ (monocytes) post	↔	↓ post	-	↔ IL-6 post	-	↔	↑ post	
								↔ IL-10 post				
								↔ IL-1ra post				
								↔ IL-8 post				
[90]	1.5 h TT running	-	8%	-	-	-	-	↓ IL-6 post	-	-	↑ post	
[91]	2 h run, followed by 5 km TT	60% VO_2max_	8%	-	-	-	-	↓ IL-6 post	-	-	↑ post	

Dosage studies were done to investigate if a higher dose (12%–12.8%) of supplemented CHO compared to a lower dose (6%–6.4%) would raise the magnitude of attenuating effects on several immunological markers [72,74]. No dose-dependent differences were found and it was concluded that ingesting at least 6% CHO beverages during exercise may sufficiently attenuate hormonal and immune responses to exercise [72,74]. Cox *et al*. [92] examined the effects of a 28-day pre-exercise high-CHO diet (8.5 g/kg BW/day) and acute CHO supplementation (10% CHO beverage) during exercise on cytokine responses following high-intensity cycling and concluded that chronic and acute CHO consumption do not have any synergistic effects on cytokine responses. In a following study, the same group showed that consuming a CHO-containing pre-exercise meal (2.1 g CHO/kg BW) may reduce the attenuating effects of CHO ingestion during exercise (10% CHO) on cytokine responses [93].

#### 2.1.3. Post-Exercise Carbohydrate Ingestion and Immune Function

Consumption of small amounts of CHO (1.0–1.2 g/kg BW) and protein immediately after exercise and during recovery is generally recommended to replenish body glycogen stores [49], to stimulate muscle protein synthesis [94] and to enhance training adaptations [95]. Very few trials addressing the influence of post-exercise CHO ingestion on immune variables after strenuous exercise exist. Ingestion of 1.2 g CHO/kg BW immediately post-exercise seems to have no attenuating effect during early recovery on total numbers of leukocytes and lymphocytes but prevented neutrophil degranulation after two hours of running at 75% VO_2max_ [96]. Plasma concentrations of IL-6 during recovery may also be unaffected when feeding 1.0 g CHO/kg BW during early recovery following cycling at 65% VO_2max_ to exhaustion [97].

### 2.2. Dietary Protein, Amino Acids and Exercise Immune Function

It is well accepted that protein deficiency impairs immune function and leads to an increased susceptibility to infection, because the production of some important immune variables, such as cytokines, immunoglobulins and acute phase proteins, depends on adequate protein availability [40,62]. The severity of protein deficiency influences the magnitude of immune system impairment [32], and protein-energy malnutrition may affect all forms of immunity [40]. Therefore, availability of adequate amounts of all amino acids is required for a maintained immuno-competence [98].

Collected data from dietary surveys of professional cyclists and elite runners revealed, that their daily intake of protein (>1.5 g/kg BW/day) easily meets the recent recommendations of daily protein intake for endurance athletes (1.2–1.7 g/kg BW/day [99]) [41]. Due to these results, protein deficiency may not be really an issue among endurance athletes but should be kept in mind when those athletes are dealing with energy-restricted dietary practices or excessive use of supplements. In exercise immunology there was slightly more interest during the last decade on specific amino acids, such as glutamine, branched chain amino acids (BCAAs) and cysteine, as well as on creatine and their possible effects on exercise immunity.

Murakami *et al*. [100,101] showed in two studies that supplementation with 700 mg cystine (dipeptid of cysteine) and 280 mg theanine (amino acid in green tea) several days prior and during a training camp, results in a significant decrease of post-exercise neutrophilia, attenuated lymphocytopenia, constant CRP levels but no differences in mucosal immunity compared to PLA. Very limited evidence exists on creatine supplementation and its effects on exercise immunity. A decreased pro-inflammatory cytokine response and lowered prostaglandin level after a half-triathlon when supplemented with a pre-event daily dosage of 20 g creatine for five days was recently reported by Bassit *et al*. [102]. Similar results after a 30 km-run were previously shown by Santos *et al*. when using the same supplementation protocol [103]. Despite some promising results, further research is needed.

#### 2.2.1. Glutamine & the “Glutamine Hypothesis”

Glutamine is the most abundant amino acid in human muscle and plasma [32]. It is a major fuel for leukocytes and lymphocytes [104] and plays an important role in protein synthesis, cytokine production and macrophage function [98]. Prolonged exercise is associated with a decreased plasma glutamine concentration by about 20% [1] and it has been hypothesized that such a substantial fall may directly lead to immunodepression (“*glutamine hypothesis*”) [32] and a higher risk for URTI [105].

Due to the attractiveness of this theory and the proven beneficial effects of glutamine in some clinical situations [106], glutamine and its effects on exercise related immune parameters has received much attention [107]. To date only one study has demonstrated a prophylactic effect of glutamine supplementation on the incidence of URTI symptoms [108]. It was reported that a significant lower incidence of URTI symptoms (32%) occurred in the 7-day period following a marathon-type event in the glutamine-supplemented group of runners (5 g glutamine in 330 mL water) compared with the placebo group.

Otherwise the majority of following studies have failed to confirm initial findings or to show beneficial effects when supplementing glutamine to maintain glutamine levels during exercise on various immune parameters, such as s-IgA levels [109,110,111], post-exercise IL-6 levels [112,113], acute phase proteins [112], lymphocyte and neutrophil counts [114] and post-exercise leukocytosis and neutrophil function [115]. Therefore, investigators have not been able to verify a direct link between decreased plasma glutamine levels and immune system changes induced by prolonged exercise [116]. 

From a practical point of view doses in excess of 5 g glutamine have to be ingested every 30–60 min during exercise to elevate plasma glutamine concentration [105], which may not be feasible in everyday training regimens. As shown by Bacurau *et al*. [71] it may be possible to prevent the exercise-induced reduction of plasma glutamine concentration by delivering an adequate amount of carbohydrate during exercise.

#### 2.2.2. Branched Chain Amino Acids

Although the BCAAs leucine, isoleucine and valine are known to have beneficial effects on reducing exercise-induced muscle damage [117] little is known about their effects on exercise immune function. Animal feeding and *in vitro* studies showed that BCAAs are necessary for efficient immune function [118], because they are used directly for protein synthesis and cytokine activation [98] or glutamine synthesis [32].

Some studies investigated the effects of pre-exercise BCAA ingestion on plasma glutamine levels and other immune variables. BCAA supplementation (6 g/day) for 30 days and an additional 3 g-dose 30 min before a triathlon inhibited exercise-induced plasma glutamine fall and modified the cytokine response to exercise [119]. Interestingly a 34% decrease in reported symptoms of infection in the BCCA supplemented group compared to PLA was observed [119]. Similar outcomes were shown in a following study comparing the effects of the same supplementation regimen on immune response in triathletes and runners [120]. However some argued that the study design was complicated and different between subject groups making the interpretation of results difficult [118,121] and thereforethese findings need to be confirmed with more controlled studies [32].

### 2.3. Dietary Fat, Fatty Acids and Exercise Immune Function

It is well established that dietary fats (amounts and composition) play a role in modulating immune functions and inflammatory processes [122]. There is some evidence that consumption of polyunsaturated fatty acids may have positive effects on some chronic diseases [123]. However, to date only a few studies have assessed how fat and fatty acids affect immune function in athletes.

#### 2.3.1. Dietary Fat Intake

Few studies have evaluated the effects of a high-fat diet (40%–62% dietary fat/day) compared to a low-fat diet (15%–19% dietary fat/day) on several aspects of post-exercise immunity [124,125,126,127]. Mainly no significant differences between the high- and low-fat diets on post-exercise lymphocyte cell counts and lymphocyte subsets [126], neutrophils and other leukocyte subsets [124] and cytokine response [124,127] were found. However, significant higher pre- and post-exercise cortisol levels [125] and decreased NK cell activity in a fat-rich diet compared to a low-fat diet [126] were shown. Some investigators argued that training on a very low-fat diet (15% dietary fat/day) may lead to an increased pro-inflammatory cytokine production [124] or an overall compromised immune function due to a negative energy balance [125] and a possible deficiency of essential micronutrients (e.g., vitamin E) [32].

#### 2.3.2. Omega-3 Polyunsaturated Fatty Acids

The essential Omega-3 (*n*-3) polyunsaturated fatty acids (PUFA) eicosapentaenoic acid (EPA) and docosahexaenoic acid (DHA), both found in oily fish and fish oils, are strong anti-inflammatory agents. Amongst other things they suppress the production of arachidonic acid, prostaglandins, and leucotrienes that modulate the production of pro-inflammatory cytokines [122,128,129]. Despite their beneficial health-related characteristics, limited evidence addressing exercise-related anti-inflammatory effects from *n*-3 PUFA supplementation exists.

Supplementation protocols varied considerably between trials and daily dosage ranged from 1.3–2.2 g EPA and 0.3–2.2 g DHA during a 4- to 6-week period before strenuous exercise [130,131,132,133,134]. Mainly no effects on post-exercise inflammatory variables or markers of oxidative stress were shown. Slight effects on cytokine milieu were revealed in only one trial when EPA and DHA were supplemented alone [130] or combined with lycopene [132]. Although dietary mixes with EPA and DHA may be beneficial in clinical trials [135], a recent study was not able to show a marked influence on post-exercise immune variables when EPA and DHA (400 mg each) were combined with other dietary immunostimulants, such as quercetin [136]. Interestingly there is a wide variance in the EPA:DHA ratio used in the presented studies, ranging from 1:4 [132] to 1:1 [133,136] and 2:1 [134] up to 4–5:1 [130,131] although general guidelines suggest an EPA:DHA ratio of 2:1 for athletes [129].

## 3. Results

Nutrient availability influences immune function in direct and indirect ways and it can be concluded that a poor nutrition state affects almost all aspects of the immune system. Otherwise it has been shown that evidence for a beneficial influence on immune parameters in athletes from single macronutrients is scarce and results are often inconsistent. Exercising in a CHO-depleted state may result in higher levels of stress hormones and an impaired immune function. This is an important issue to consider in view of new training strategies that involve training with low glycogen or CHO availability. These are very popular nowadays, because there is some evidence that it may enhance the training response [49].

There is some evidence that frequent ingestion of a ≥6% CHO solution (typically sport drinks) during prolonged exercise maintains blood glucose level and may help to attenuate exercise induced changes of stress hormone levels, leukocyte cell counts and cytokine changes, whereas it is possible that the attenuating effects may be reduced by a pre-exercise CHO containing meal. Post-exercise feeding of CHO seems to have no beneficial effect on changes in immune function.

Protein deficiency may not really be an issue in endurance sports, as cyclists and runners easily meet their protein demands [41], but should be kept in mind for those athletes who are on energy-restricted diets or consuming supplements. Although there are some promising results from studies on the effects creatine or cystine/theanine supplementation on immune function in athletes, further research is needed. Glutamine plays an important role in immunity, yet there is currently no evidence to support the use of glutamine supplements to enhance immune function in athletes [105]. BCAAs, precursors of glutamine, may have some immunomodulating effects, but strong evidence is still outstanding.

Cyclists and runners desire low body fat and leanness for optimal performance and therefore often follow energy- and/or fat-restricted diets [41].Training on a very low-fat diet (15% dietary fat) may be detrimental to exercise performance and leads to an overall compromised immune function due to a negative energy balance [125] and micronutrient deficiency [32]. High-fat diets (≥40% dietary fat) have also been suggested to be detrimental to the immune system [126]. Although *n*-3 PUFA are essential to the athlete’s health [129] and are known to be strong anti-inflammatory agents, no beneficial effects of fish oil supplementation on the immunological response to strenuous exercise have been shown. Thus, athletes are advised to follow general recommendations of dietary fat intake without an excessive supplementation of their diet with *n*-3 PUFA, because they are also known to be immunosuppressive [32]. Table 3 depicts the immunomodulating nutritional strategies and countermeasures presented in this paper and the evidence and likely impact of the underlying rationale respectively.

**Table 3:** Immunomodulating nutritional strategies & countermeasures: evidence and likely impact (CHO: carbohydrate; BCAA: branched chain amino acid; *n*-3 PUFA: Omega-3 polyunsaturated fatty acids; evidence for rationale: −: no evidence; +: very limited evidence exists—more research is needed; ++: limited evidence exists—more research is needed; +++: relatively strong evidence; ++++: strong evidence; likely impact: −: no influence; +: very limited influence; ++: limited influence; +++: relatively strong influence; ++++: strong influence).

Nutrient/Strategy	Rationale	Evidence	Likely Impact
Adequate nutrient availability (e.g., micronutrients, fluid)	Adequate nutrient availability maintains immunocompetence	++++	++++
High-CHO diet	Maintained blood glucose level → lower stress hormone levels → attenuated post-ex immune response	++	++
CHO ingestion during exercise	Maintained blood glucose level → lower stress hormone levels → attenuated post-ex immune response	+++	+++
CHO ingestion post-exercise	Attenuating effect on some immune variables (prevents lymphocytopenia, faster IL-6 return to pre-exercise level) during recovery	−	−
Dietary protein availability	Protein is needed for production of immune variables	++	++
Glutamine	Glutamine hypothesis; protein synthesis	−	+
BCAA	Precursors of glutamine	++	+
Creatine	Muscle trauma from heavy exercise → higher inflammatory markers (TNF-α, prostaglandin).	+	+
	Creatine prevents muscle trauma → attenuated inflammation markers		
Cystine/theanine	Reinforced glutathione synthesis → reinforced anti-oxidative response & better immune function	+	+
Dietary fat intake	Low-fat: energy & micronutrient deficiency	++	++
	High-fat: excessive intake at cost of protein/CHO		
*n*-3 PUFA	Anti-inflammatory effects of *n*-3 PUFA	−	−

## 4. Discussion & Future Perspectives

Numerous attempts have been made to attenuate exercise-induced immune cell perturbations with single nutrients. Evidence for a beneficial influence on immune parameters in athletes from single macronutrients is scarce and results are often inconsistent. Only when carbohydrates are frequently delivered during prolonged exercise may an influence on the immune response to exercise to a larger or smaller extent be possible. To date no other effective approaches exist and no explicit nutritional recommendations to influence the immunological response to high intensity exercise or to reduce post-exercise URTI symptoms can be derived.

The large number of “negative” findings on the effects of nutritional supplements to prevent immunosuppression in athletes may be due to multiple influencing factors. The lack of quality of many of the reviewed trials introduces wide variation in results, and makes it difficult to compare the results of different trials. Larger trials with uniform endpoints are necessary [137]. Despite evidence from clinical trials, some nutritional supplements (e.g., glutamine, *n*-3 PUFA) seem to be ineffective in modulating exercise-induced immune changes. One reason for this disparity is that because the immune system is so diverse, using a single nutrient may not be as effective as a combination of nutrients [4]. Nutritional supplements should improve innate immunity, which provides host protection against a wide variety of pathogens. The risk of infection can be more effectively decreased when innate immunity is enhanced than when the slower adaptive immunity is targeted [4]. Dosage and time of nutrient ingestion may also play an influential role. Further, it has been shown that CHO supplementation attenuates the IL-6 response to exercise. Petersen & Pedersen [138] argued that IL-6 has some potential anti-inflammatory and metabolic effects. Inhibited release of IL-6 lowers the anti-inflammatory cytokine response, inhibits lypolysis, which is rather a desired effect of exercising, and may reduce training adaptation [16]. Therefore, attenuation of IL-6 might not be desired—the debate in scientific literature is still going on. Exercise-induced immunodepression in athletes is typically transient and some investigators argued that it might be a necessary form of adaptation to training [22,26] and questioned its clinical relevance [137].

Despite many unresolved issues on this topic, attention has been recently drawn to investigate potential beneficial effects of dietary immunostimulants, such as bovine colostrum [139,140,141], probiotics [142,143,144], β-glucans [145,146,147,148] or anti-oxidants [137,149,150]—mainly with inconsistent results and still without strong evidence.

An overall adequate nutrient availability provided by a well-balanced diet and sufficient fluid delivery may help to maintain immunocompetence in athletes, since inadequate nutrition affects almost all aspects of the immune system. In the expanding field of exercise immunology much has been done, but there is still a great deal more to learn. The ultimate goal of future research is to create a sports drink that contains carbohydrate and a cocktail of immunomodulatory supplements that attenuate markers of inflammation and reduce the risk of infection [4].

## References

[1] Jeukendrup A.E., Gleeson M. (2010). Sport Nutrition: An Introduction to Energy Production and Performance.

[2] Gleeson M., Nieman D.C., Pedersen B.K. (2004). Exercise, nutrition and immune function. J. Sports Sci..

[3] Maughan R.J., Gleeson M. (2010). The Biochemical Basis of Sports Performance.

[4] Walsh N.P., Gleeson M., Pyne D.B., Nieman D.C., Dhabhar F.S., Shephard R.J., Oliver S.J., Bermon S., Kajeniene A. (2011). Position statement. Part two: Maintaining immune health. Exerc. Immunol. Rev..

[5] Peters E.M., Bateman E.D. (1983). Ultramarathon running and upper respiratory tract infections. An epidemiological survey. S. Afr. Med. J..

[6] Nieman D.C., Johanssen L.M., Lee J.W. (1989). Infectious episodes in runners before and after a roadrace. J. Sports Med. Phys. Fitness.

[7] Nieman D.C., Johanssen L.M., Lee J.W., Arabatzis K. (1990). Infectious episodes in runners before and after the Los Angeles Marathon. J. Sports Med. Phys. Fitness.

[8] Heath G.W., Ford E.S., Craven T.E., Macera C.A., Jackson K.L., Pate R.R. (1991). Exercise and the incidence of upper respiratory tract infections. Med. Sci. Sports Exerc..

[9] Peters E.M., Goetzsche J.M., Grobbelaar B., Noakes T.D. (1993). Vitamin C supplementation reduces the incidence of postrace symptoms of upper-respiratory-tract infection in ultramarathon runners. Am. J. Clin. Nutr..

[10] Nieman D.C. (2009). Immune function responses to ultramarathon race competition. Med. Sport..

[11] Gleeson M., Bishop N.C., Oliveira M., Tauler P. (2011). Influence of training load on upper respiratory tract infection incidence and antigen-stimulated cytokine production. Scand. J. Med. Sci. Sports.

[12] Ekblom B., Ekblom O., Malm C. (2006). Infectious episodes before and after a marathon race. Scand. J. Med. Sci. Sports.

[13] Pacqué P.F., Booth C., Ball M., Dwyer D. (2007). The effect of an ultra-endurance running race on mucosal and humoral immune function. J. Sports Med. Phys. Fitness.

[14] Nieman D.C., Pedersen B.K. (1999). Exercise and immune function. Recent developments. Sports Med..

[15] Walsh N.P., Gleeson M., Shephard R.J., Gleeson M., Woods J.A., Bishop N.C., Fleshner M., Green C., Pedersen B.K., Hoffman-Goetz L. (2011). Position statement. Part one: Immune function and exercise. Exerc. Immunol. Rev..

[16] Gleeson M. (2007). Immune function in sport and exercise. J. Appl. Physiol..

[17] Brolinson P.G., Elliott D. (2007). Exercise and the immune system. Clin. Sports Med..

[18] Nieman D.C. (1997). Risk of upper respiratory tract infection in athletes: An epidemiologic and immunologic perspective. J. Athl. Train..

[19] Bishop N.C., Gleeson M. (2006). Exercise and Infection Risk. Immune Function in Sport and Exercise. Advances in Sport and Exercise Science Series.

[20] Moreira A., Delgado L., Moreira P., Haahtela T. (2009). Does exercise increase the risk of upper respiratory tract infections?. Br. Med. Bull..

[21] Schwellnus M., Lichaba M., Derman E. (2010). Respiratory tract symptoms in endurance athletes—A review of causes and consequences. Curr. Allergy Clin. Immunol..

[22] Peters E.M. (2004). Postrace upper respiratory tract ‘infections’ in ultramarathoners—Infection, allergy or inflammation?. S. Afr. J. Sports Med..

[23] Nieman D.C., Henson D.A., Austin M.D., Sha W. (2011). Upper respiratory tract infection is reduced in physically fit and active adults. Br. J. Sports Med..

[24] Nieman D.C. (2000). Special feature for the Olympics: Effects of exercise on the immune system: Exercise effects on systemic immunity. Immunol. Cell Biol..

[25] Albers R., Antoine J.M., Bourdet-Sicard R., Calder P.C., Gleeson M., Lesourd B., Samartin S., Sanderson I.R., van Loo J., Vas Dias F.W. (2005). Markers to measure immunomodulation in human nutrition intervention studies. Br. J. Nutr..

[26] Nieman D.C. (2008). Immunonutrition support for athletes. Nutr. Rev..

[27] Blannin A.K., Gleeson M. (2006). Acute Exercise and Innate Immune Function. Immune Function in Sport and Exercise. Advances in Sport and Exercise Science Series.

[28] Timmons B.W., Tarnopolsky M.A., Snider D.P., Bar-Or O. (2006). Immunological changes in response to exercise: Influence of age, puberty, and gender. Med. Sci. Sports Exerc..

[29] Pedersen B.K., Hoffman-Goetz L. (2000). Exercise and the immune system: Regulation, integration, and adaptation. Physiol. Rev..

[30] Bishop N.C., Gleeson M. (2006). Acute Exercise and Aquired Immune Function. Immune Function in Sport and Exercise. Advances in Sport and Exercise Science Series.

[31] Cooper D.M., Radom-Aizik S., Schwindt C., Zaldivar F. (2007). Dangerous exercise: Lessons learned from dysregulated inflammatory responses to physical activity. J. Appl. Physiol..

[32] Walsh N.P., Gleeson M. (2006). Exercise, Nutrition and Immune Function. I. Macronutrients and Amino Acids. Immune Function in Sport and Exercise. Advances in Sport and Exercise Science Series.

[33] Gleeson M., Robson-Ansley P., Gleeson M. (2006). Immune Response to Intensified Training and Overtraining. Immune Function in Sport and Exercise. Advances in Sport and Exercise Science Series.

[34] MacKinnon L.T. (2000). Special feature for the Olympics: Effects of exercise on the immune system: Overtraining effects on immunity and performance in athletes. Immunol. Cell Biol..

[35] Fahlman M.M., Engels H.J. (2005). Mucosal IgA and URTI in American college football players: A year longitudinal study. Med. Sci. Sports Exerc..

[36] Lancaster G.I., Halson S.L., Khan Q., Drysdale P., Jeukendrup A.E., Drayson M.T., Gleeson M. (2004). Effects of acute exhaustive exercise and chronic exercise training on type 1 and type 2 T lymphocytes. Exerc. Immunol. Rev..

[37] Gleeson M., McDonald W.A., Cripps A.W., Pyne D.B., Clancy R.L., Fricker P.A. (1995). The effect on immunity of long-term intensive training in elite swimmers. Clin. Exp. Immunol..

[38] Ronsen O., Kjeldsen-Kragh J., Haug E., Bahr R., Pedersen B.K. (2002). Recovery time affects immunoendocrine responses to a second bout of endurance exercise. Am. J. Physiol. Cell Physiol..

[39] Degerstrom J., Osterud B. (2006). Increased inflammatory response of blood cells to repeated bout of endurance exercise. Med. Sci. Sports Exerc..

[40] Calder P.C., Jackson A.A. (2000). Undernutrition, infection and immune function. Nutr. Res. Rev..

[41] Burke L. (2007). Practical Sports Nutrition.

[42] Calder P.C. Immunodepression and Exercise: The Evidence & an Evaluation of Preventive Nutritional Strategies. Exercise and Immunity in Athletic Performance and a Healthy Life, Proceedings of the 10th ISEI Symposium.

[43] Pyne D.B., Burke L., Deakin V. (2006). Nutrition for the Athlete’s Immune System: Eating to Stay Well during Training and Competition. Clinical Sports Nutrition.

[44] Scott J.P., Sale C., Greeves J.P., Casey A., Dutton J., Fraser W.D. (2011). Effect of exercise intensity on the cytokine response to an acute bout of running. Med. Sci. Sports Exerc..

[45] Nieman D.C., Konrad M., Henson D.A., Kennerly K., Shanely R.A., Wallner-Liebmann S.J. (2012). Variance in the acute inflammatory response to prolonged cycling is linked to exercise intensity. J. Interferon Cytokine Res..

[46] Peake J.M., Suzuki K., Hordern M., Wilson G., Nosaka K., Coombes J.S. (2005). Plasma cytokine changes in relation to exercise intensity and muscle damage. Eur. J. Appl. Physiol..

[47] Gleeson M., Bishop N.C. (2000). Special feature for the Olympics: Effects of exercise on the immune system: Modification of immune responses to exercise by carbohydrate, glutamine and anti-oxidant supplements. Immunol. Cell Biol..

[48] Nieman D.C. (2011). Exercise Testing and Prescription: A Health-Related Approach.

[49] Burke L.M. (2010). Fueling strategies to optimize performance: Training high or training low?. Scand. J. Med. Sci. Sports.

[50] Nieman D.C., Henson D.A., Smith L.L., Utter A.C., Vinci D.M., Davis J.M., Kaminsky D.E., Shute M. (2001). Cytokine changes after a marathon race. J. Appl. Physiol..

[51] Mitchell J.B., Pizza F.X., Paquet A., Davis B.J., Forrest M.B., Braun W.A. (1998). Influence of carbohydrate status on immune responses before and after endurance exercise. J. Appl. Physiol..

[52] Gleeson M., Blannin A.K., Walsh N.P., Bishop N.C., Clark A.M. (1998). Effect of low- and high-carbohydrate diets on the plasma glutamine and circulating leukocyte responses to exercise. Int. J. Sport Nutr..

[53] Bishop N.C., Walsh N.P., Haines D.L., Richards E.E., Gleeson M. (2001). Pre-Exercise carbohydrate status and immune responses to prolonged cycling: I. Effect on neutrophil degranulation. Int. J. Sport Nutr. Exerc. Metab..

[54] Bishop N.C., Walsh N.P., Haines D.L., Richards E.E., Gleeson M. (2001). Pre-Exercise carbohydrate status and immune responses to prolonged cycling: II. Effect on plasma cytokine concentration. Int. J. Sport Nutr. Exerc. Metab..

[55] Close G.L., Ashton T., Cable T., Doran D., Noyes C., McArdle F., MacLaren D.P. (2005). Effects of dietary carbohydrate on delayed onset muscle soreness and reactive oxygen species after contraction induced muscle damage. Br. J. Sports Med..

[56] Costa R.J., Jones G.E., Lamb K.L., Coleman R., Williams J.H. (2005). The effects of a high carbohydrate diet on cortisol and salivary immunoglobulin A (s-IgA) during a period of increase exercise workload amongst Olympic and Ironman triathletes. Int. J. Sports Med..

[57] Blanchard M.A., Jordan G., Desbrow B., MacKinnon L.T., Jenkins D.G. (2001). The influence of diet and exercise on muscle and plasma glutamine concentrations. Med. Sci. Sports Exerc..

[58] De Sousa M.V., Madsen K., Simoes H.G., Pereira R.M., Negrao C.E., Mendonca R.Z., Takayama L., Fukui R., da Silva M.E. (2010). Effects of carbohydrate supplementation on competitive runners undergoing overload training followed by a session of intermittent exercise. Eur. J. Appl. Physiol..

[59] Chen Y.J., Wong S.H., Wong C.K., Lam C.W., Huang Y.J., Siu P.M. (2008). The effect of a pre-exercise carbohydrate meal on immune responses to an endurance performance run. Br. J. Nutr..

[60] Chen Y.J., Wong S.H., Chan C.O., Wong C.K., Lam C.W., Siu P.M. (2009). Effects of glycemic index meal and CHO-electrolyte drink on cytokine response and run performance in endurance athletes. J. Sci. Med. Sport.

[61] Li T.L., Wu C.L., Gleeson M., Williams C. (2004). The effects of pre-exercise high carbohydrate meals with different glycemic indices on blood leukocyte redistribution, IL-6, and hormonal responses during a subsequent prolonged exercise. Int. J. Sport Nutr. Exerc. Metab..

[62] Gleeson M. (2006). Can nutrition limit exercise-induced immunodepression?. Nutr. Rev..

[63] Nehlsen-Cannarella S.L., Fagoaga O.R., Nieman D.C., Henson D.A., Butterworth D.E., Schmitt R.L., Bailey E.M., Warren B.J., Utter A., Davis J.M. (1997). Carbohydrate and the cytokine response to 2.5 h of running. J. Appl. Physiol..

[64] Nieman D.C., Fagoaga O.R., Butterworth D.E., Warren B.J., Utter A., Davis J.M., Henson D.A., Nehlsen-Cannarella S.L. (1997). Carbohydrate supplementation affects blood granulocyte and monocyte trafficking but not function after 2.5 h or running. Am. J. Clin. Nutr..

[65] Nieman D.C., Henson D.A., Garner E.B., Butterworth D.E., Warren B.J., Utter A., Davis J.M., Fagoaga O.R., Nehlsen-Cannarella S.L. (1997). Carbohydrate affects natural killer cell redistribution but not activity after running. Med. Sci. Sports Exerc..

[66] Henson D.A., Nieman D.C., Nehlsen-Cannarella S.L., Fagoaga O.R., Shannon M., Bolton M.R., Davis J.M., Gaffney C.T., Kelln W.J., Austin M.D. (2000). Influence of carbohydrate on cytokine and phagocytic responses to 2 h of rowing. Med. Sci. Sports Exerc..

[67] Bishop N.C., Walker G.J., Bowley L.A., Evans K.F., Molyneux K., Wallace F.A., Smith A.C. (2005). Lymphocyte responses to influenza and tetanus toxoid *in vitro* following intensive exercise and carbohydrate ingestion on consecutive days. J. Appl. Physiol..

[68] Peake J., Peiffer J.J., Abbiss C.R., Nosaka K., Laursen P.B., Suzuki K. (2008). Carbohydrate gel ingestion and immunoendocrine responses to cycling in temperate and hot conditions. Int. J. Sport Nutr. Exerc. Metab..

[69] Henson D.A., Nieman D.C., Blodgett A.D., Butterworth D.E., Utter A., Davis J.M., Sonnenfeld G., Morton D.S., Fagoaga O.R., Nehlsen-Cannarella S.L. (1999). Influence of exercise mode and carbohydrate on the immune response to prolonged exercise. Int. J. Sport Nutr..

[70] Nieman D.C., Davis J.M., Henson D.A., Walberg-Rankin J., Shute M., Dumke C.L., Utter A.C., Vinci D.M., Carson J.A., Brown A. (2003). Carbohydrate ingestion influences skeletal muscle cytokine mRNA and plasma cytokine levels after a 3-h run. J. Appl. Physiol..

[71] Timmons B.W., Tarnopolsky M.A., Bar-Or O. (2004). Immune responses to strenuous exercise and carbohydrate intake in boys and men. Pediatr. Res..

[72] Lancaster G.I., Khan Q., Drysdale P.T., Wallace F., Jeukendrup A.E., Drayson M.T., Gleeson M. (2005). Effect of prolonged exercise and carbohydrate ingestion on type 1 and type 2 T lymphocyte distribution and intracellular cytokine production in humans. J. Appl. Physiol..

[73] Walker G.J., Finlay O., Griffiths H., Sylvester J., Williams M., Bishop N.C. (2007). Immunoendocrine response to cycling following ingestion of caffeine and carbohydrate. Med. Sci. Sports Exerc..

[74] Scharhag J., Meyer T., Auracher M., Gabriel H.H., Kindermann W. (2006). Effects of graded carbohydrate supplementation on the immune response in cycling. Med. Sci. Sports Exerc..

[75] Green K.J., Croaker S.J., Rowbottom D.G. (2003). Carbohydrate supplementation and exercise-induced changes in T-lymphocyte function. J. Appl. Physiol..

[76] Henson D.A., Nieman D.C., Pistilli E.E., Schilling B., Colacino A., Utter A.C., Fagoaga O.R., Vinci D.M., Nehlsen-Cannarella S.L. (2004). Influence of carbohydrate and age on lymphocyte function following a marathon. Int. J. Sport Nutr. Exerc. Metab..

[77] McFarlin B.K., Flynn M.G., Stewart L.K., Timmerman K.L. (2004). Carbohydrate intake during endurance exercise increases natural killer cell responsiveness to IL-2. J. Appl. Physiol..

[78] Nieman D.C., Henson D.A., Gojanovich G., Davis J.M., Murphy E.A., Mayer E.P., Pearce S., Dumke C.L., Utter A.C., McAnulty S.R. (2006). Influence of carbohydrate on immune function following 2 h cycling. Res. Sports Med..

[79] Gleeson M., Gleeson M. (2006). Introduction to the Immune System. Immune Function in Sport and Exercise. Advances in Sport and Exercise Science Series.

[80] Li T.L., Gleeson M. (2005). The effects of carbohydrate supplementation during repeated bouts of prolonged exercise on saliva flow rate and immunoglobulin A. J. Sports Sci..

[81] Bishop N.C., Blannin A.K., Armstrong E., Rickman M., Gleeson M. (2000). Carbohydrate and fluid intake affect the saliva flow rate and IgA response to cycling. Med. Sci. Sports Exerc..

[82] Nieman D.C., Nehlsen-Cannarella S.L., Fagoaga O.R., Henson D.A., Utter A., Davis J.M., Williams F., Butterworth D.E. (1998). Effects of mode and carbohydrate on the granulocyte and monocyte response to intensive, prolonged exercise. J. Appl. Physiol..

[83] Starkie R.L., Arkinstall M.J., Koukoulas I., Hawley J.A., Febbraio M.A. (2001). Carbohydrate ingestion attenuates the increase in plasma interleukin-6, but not skeletal muscle interleukin-6 mRNA, during exercise in humans. J. Physiol..

[84] Bacurau R.F., Bassit R.A., Sawada L., Navarro F., Martins E., Costa Rosa L.F. (2002). Carbohydrate supplementation during intense exercise and the immune response of cyclists. Clin. Nutr..

[85] Bishop N.C., Gleeson M., Nicholas C.W., Ali A. (2002). Influence of carbohydrate supplementation on plasma cytokine and neutrophil degranulation responses to high intensity intermittent exercise. Int. J. Sport Nutr. Exerc. Metab..

[86] Febbraio M.A., Steensberg A., Keller C., Starkie R.L., Nielsen H.B., Krustrup P., Ott P., Secher N.H., Pedersen B.K. (2003). Glucose ingestion attenuates interleukin-6 release from contracting skeletal muscle in humans. J. Physiol..

[87] Bishop N.C., Walsh N.P., Scanlon G.A. (2003). Effect of prolonged exercise and carbohydrate on total neutrophil elastase content. Med. Sci. Sports Exerc..

[88] Nieman D.C., Davis J.M., Henson D.A., Gross S.J., Dumke C.L., Utter A.C., Vinci D.M., Carson J.A., Brown A., McAnulty S.R. (2005). Muscle cytokine mRNA changes after 2.5 h of cycling: Influence of carbohydrate. Med. Sci. Sports Exerc..

[89] Mamus R.T., Dos Santos M.G., Campbell B., Kreider R. (2006). Biochemical effects of carbohydrate supplementation in a simulated competition of short terrestrial duathlon. J. Int. Soc. Sports Nutr..

[90] Robson-Ansley P., Barwood M., Eglin C., Ansley L. (2009). The effect of carbohydrate ingestion on the interleukin-6 response to a 90-minute run time trial. Int. J. Sports Physiol. Perform..

[91] Robson-Ansley P., Walshe I., Ward D. (2011). The effect of carbohydrate ingestion on plasma interleukin-6, hepcidin and iron concentrations following prolonged exercise. Cytokine.

[92] Cox A.J., Pyne D.B., Cox G.R., Callister R., Gleeson M. Effect of Chronic Carbohydrate Consumption on Cytokine Responses to Cycle Ergometry. Inflammation in Exercise: Friend or Foe? Proceeding of the 8th International Society of Exercise and Immunology Symposium.

[93] Cox A.J., Pyne D.B., Cox G.R., Callister R., Gleeson M. (2008). Pre-Exercise carbohydrate status influences carbohydrate-mediated attenuation of post-exercise cytokine responses. Int. J. Sports Med..

[94] Kerksick C., Harvey T., Stout J., Campbell B., Wilborn C., Kreider R., Kalman D., Ziegenfuss T., Lopez H., Landis J. (2008). International society of sports nutrition position stand: Nutrient timing. J. Int. Soc. Sports Nutr..

[95] Ferguson-Stegall L., McCleave E., Ding Z., Doerner P.G., Liu Y., Wang B., Healy M., Kleinert M., Dessard B., Lassiter D.G. (2011). Aerobic exercise training adaptations are increased by postexercise carbohydrate-protein supplementation. J. Nutr. Metab..

[96] Costa R.J., Walters R., Bilzon J.L., Walsh N.P. (2011). Effects of immediate postexercise carbohydrate ingestion with and without protein on neutrophil degranulation. Int. J. Sport Nutr. Exerc. Metab..

[97] Gusba J.E., Wilson R.J., Robinson D.L., Graham T.E. (2008). Interleukin-6 and its mRNA responses in exercise and recovery: Relationship to muscle glycogen. Scand. J. Med. Sci. Sports.

[98] Li P., Yin Y.L., Li D., Kim S.W., Wu G. (2007). Amino acids and immune function. Br. J. Nutr..

[99] Rodriguez N.R., Di Marco N.M., Langley S., American Dietetic Association, Dietitians of Canada, American College of Sports Medicine (2009). American college of sports medicine position stand. Nutrition and athletic performance. Med. Sci. Sports Exerc..

[100] Murakami S., Kurihara S., Koikawa N., Nakamura A., Aoki K., Yosigi H., Sawaki K., Ohtani M. (2009). Effects of oral supplementation with cystine and theanine on the immune function of athletes in endurance exercise: Randomized, double-blind, placebo-controlled trial. Biosci. Biotechnol. Biochem..

[101] Murakami S., Kurihara S., Titchenal C.A., Ohtani M. (2010). Suppression of exercise-induced neutrophilia and lymphopenia in athletes by cystine/theanine intake: A randomized, double-blind, placebo-controlled trial. J. Int. Soc. Sports Nutr..

[102] Bassit R.A., Curi R., Costa Rosa L.F. (2008). Creatine supplementation reduces plasma levels of pro-inflammatory cytokines and PGE2 after a half-ironman competition. Amino Acids.

[103] Santos R.V., Bassit R.A., Caperuto E.C., Costa Rosa L.F. (2004). The effect of creatine supplementation upon inflammatory and muscle soreness markers after a 30km race. Life Sci..

[104] Braun W.A., von Duvillard S.P. (2004). Influence of carbohydrate delivery on the immune response during exercise and recovery from exercise. Nutrition.

[105] Gleeson M. (2008). Dosing and efficacy of glutamine supplementation in human exercise and sport training. J. Nutr..

[106] Castell L.M. (2002). Can glutamine modify the apparent immunodepression observed after prolonged, exhaustive exercise?. Nutrition.

[107] Nieman D.C. (2007). Marathon training and immune function. Sports Med..

[108] Castell L.M., Poortmans J.R., Newsholme E.A. (1996). Does glutamine have a role in reducing infections in athletes?. Eur. J. Appl. Physiol. Occup. Physiol..

[109] Krzywkowski K., Petersen E.W., Ostrowski K., Link-Amster H., Boza J., Halkjaer-Kristensen J., Pedersen B.K. (2001). Effect of glutamine and protein supplementation on exercise-induced decreases in salivary IgA. J. Appl. Physiol..

[110] Roshan V.D., Barzegarzadeh H. (2009). The effect of the short-term glutamine supplementation on exhaustive exercise-induced changes in immune system of active boys. World J. Sport Sci..

[111] Krieger J.W., Crowe M., Blank S.E. (2004). Chronic glutamine supplementation increases nasal but not salivary IgA during 9 days of interval training. J. Appl. Physiol..

[112] Hoffman J.R., Ratamess N.A., Kang J., Rashti S.L., Kelly N., Gonzalez A.M., Stec M., Anderson S., Bailey B.L., Yamamoto L.M. (2010). Examination of the efficacy of acute L-alanyl-L-glutamine ingestion during hydration stress in endurance exercise. J. Int. Soc. Sports Nutr..

[113] Hiscock N., Petersen E.W., Krzywkowski K., Boza J., Halkjaer-Kristensen J., Pedersen B.K. (2003). Glutamine supplementation further enhances exercise-induced plasma IL-6. J. Appl. Physiol..

[114] Krzywkowski K., Petersen E.W., Ostrowski K., Kristensen J.H., Boza J., Pedersen B.K. (2001). Effect of glutamine supplementation on exercise-induced changes in lymphocyte function. Am. J. Physiol. Cell Physiol..

[115] Walsh N.P., Blannin A.K., Bishop N.C., Robson P.J., Gleeson M. (2000). Effect of oral glutamine supplementation on human neutrophil lipopolysaccharide-stimulated degranulation following prolonged exercise. Int. J. Sport Nutr. Exerc. Metab..

[116] Hiscock N., Pedersen B.K. (2002). Exercise-Induced immunodepression—plasma glutamine is not the link. J. Appl. Physiol..

[117] Negro M., Giardina S., Marzani B., Marzatico F. (2008). Branched-Chain amino acid supplementation does not enhance athletic performance but affects muscle recovery and the immune system. J. Sports Med. Phys. Fit..

[118] Calder P.C. (2006). Branched-Chain amino acids and immunity. J. Nutr..

[119] Bassit R.A., Sawada L.A., Bacurau R.F., Navarro F., Costa Rosa L.F. (2000). The effect of BCAA supplementation upon the immune response of triathletes. Med. Sci. Sports Exerc..

[120] Bassit R.A., Sawada L.A., Bacurau R.F., Navarro F., Martins E., Santos R.V., Caperuto E.C., Rogeri P., Costa Rosa L.F. (2002). Branched-Chain amino acid supplementation and the immune response of long-distance athletes. Nutrition.

[121] Gleeson M. (2005). Interrelationship between physical activity and branched-chain amino acids. J. Nutr..

[122] Galli C., Calder P.C. (2009). Effects of fat and fatty acid intake on inflammatory and immune responses: A critical review. Ann. Nutr. Metab..

[123] Wall R., Ross R.P., Fitzgerald G.F., Stanton C. (2010). Fatty acids from fish: The anti-inflammatory potential of long-chain omega-3 fatty acids. Nutr. Rev..

[124] Meksawan K., Venkatraman J.T., Awad A.B., Pendergast D.R. (2004). Effect of dietary fat intake and exercise on inflammatory mediators of the immune system in sedentary men and women. J. Am. Coll. Nutr..

[125] Venkatraman J.T., Feng X., Pendergast D. (2001). Effects of dietary fat and endurance exercise on plasma cortisol, prostaglandin E2, interferon-gamma and lipid peroxides in runners. J. Am. Coll. Nutr..

[126] Pedersen B.K., Helge J.W., Richter E.A., Rohde T., Kiens B. (2000). Training and natural immunity: Effects of diets rich in fat or carbohydrate. Eur. J. Appl. Physiol..

[127] Venkatraman J.T., Pendergast D. (1998). Effects of the level of dietary fat intake and endurance exercise on plasma cytokines in runners. Med. Sci. Sports Exerc..

[128] Calder P.C. (2006). *n*-3 polyunsaturated fatty acids, inflammation, and inflammatory diseases. Am. J. Clin. Nutr..

[129] Simopoulos A.P. (2007). Omega-3 fatty acids and athletics. Curr. Sports Med. Rep..

[130] Gray P., Gabriel B., Thies F., Gray S.R. (2012). Fish oil supplementation augments post-exercise immune function in young males. Brain Behav. Immun..

[131] Nieman D.C., Henson D.A., McAnulty S.R., Jin F., Maxwell K.R. (2009). *n*-3 polyunsaturated fatty acids do not alter immune and inflammation measures in endurance athletes. Int. J. Sport Nutr. Exerc. Metab..

[132] Callister R., Plunkett B., Garg M. Effects of Fish Oil and Lycopene Supplements on Cytokine Response to Exercise. Exercise Immunology—Emerging Relevance in Clinical Medicine, Proceeding of 9th Symposium of the International Society of Exercise and Immunology.

[133] Bloomer R.J., Larson D.E., Fisher-Wellman K.H., Galpin A.J., Schilling B.K. (2009). Effect of eicosapentaenoic and docosahexaenoic acid on resting and exercise-induced inflammatory and oxidative stress biomarkers: A randomized, placebo controlled, cross-over study. Lipids Health Dis..

[134] Toft A.D., Thorn M., Ostrowski K., Asp S., Moller K., Iversen S., Hermann C., Sondergaard S.R., Pedersen B.K. (2000). *n*-3 polyunsaturated fatty acids do not affect cytokine response to strenuous exercise. J. Appl. Physiol..

[135] Bakker G.C., van Erk M.J., Pellis L., Wopereis S., Rubingh C.M., Cnubben N.H., Kooistra T., van Ommen B., Hendriks H.F. (2010). An antiinflammatory dietary mix modulates inflammation and oxidative and metabolic stress in overweight men: a nutrigenomics approach. Am. J. Clin. Nutr..

[136] Konrad M., Nieman D.C., Henson D.A., Kennerly K.M., Jin F., Wallner-Liebmann S.J. (2011). The acute effect of ingesting a quercetin-based supplement on exercise-induced inflammation and immune changes in runners. Int. J. Sport Nutr. Exerc. Metab..

[137] Moreira A., Kekkonen R.A., Delgado L., Fonseca J., Korpela R., Haahtela T. (2007). Nutritional modulation of exercise-induced immunodepression in athletes: A systematic review and meta-analysis. Eur. J. Clin. Nutr..

[138] Petersen A.M., Pedersen B.K. (2005). The anti-inflammatory effect of exercise. J. Appl. Physiol..

[139] Davison G., Diment B.C. (2010). Bovine colostrum supplementation attenuates the decrease of salivary lysozyme and enhances the recovery of neutrophil function after prolonged exercise. Br. J. Nutr..

[140] Shing C.M., Peake J., Suzuki K., Okutsu M., Pereira R., Stevenson L., Jenkins D.G., Coombes J.S. (2007). Effects of bovine colostrum supplementation on immune variables in highly trained cyclists. J. Appl. Physiol..

[141] Brinkworth G.D., Buckley J.D. (2003). Concentrated bovine colostrum protein supplementation reduces the incidence of self-reported symptoms of upper respiratory tract infection in adult males. Eur. J. Nutr..

[142] West N.P., Pyne D.B., Peake J.M., Cripps A.W. (2009). Probiotics, immunity and exercise: A review. Exerc. Immunol. Rev..

[143] Gleeson M., Bishop N.C., Oliveira M., Tauler P. (2011). Daily probiotic’s (*Lactobacillus casei* Shirota) reduction of infection incidence in athletes. Int. J. Sport Nutr. Exerc. Metab..

[144] West N.P., Pyne D.B., Cripps A.W., Hopkins W.G., Eskesen D.C., Jairath A., Christophersen C.T., Conlon M.A., Fricker P.A. (2011). *Lactobacillus fermentum* (PCC^®^) supplementation and gastrointestinal and respiratory-tract illness symptoms: A randomised control trial in athletes. Nutr. J..

[145] Talbott S., Talbott J. (2009). Effect of BETA 1,3/1,6 GLUCAN on upper respiratory tract infection symptoms and mood state in marathon athletes. J. Sports Sci. Med..

[146] Nieman D.C., Henson D.A., McMahon M., Wrieden J.L., Davis J.M., Murphy E.A., Gross S.J., McAnulty L.S., Dumke C.L. (2008). Beta-Glucan, immune function, and upper respiratory tract infections in athletes. Med. Sci. Sports Exerc..

[147] Murphy E.A., Davis J.M., Brown A.S., Carmichael M.D., Ghaffar A., Mayer E.P. (2007). Oat beta-glucan effects on neutrophil respiratory burst activity following exercise. Med. Sci. Sports Exerc..

[148] Davis J.M., Murphy E.A., Brown A.S., Carmichael M.D., Ghaffar A., Mayer E.P. (2004). Effects of moderate exercise and oat beta-glucan on innate immune function and susceptibility to respiratory infection. Am. J. Physiol. Regul. Integr. Comp. Physiol..

[149] McAnulty S.R., Nieman D.C., McAnulty L.S., Lynch W.S., Jin F., Henson D.A. (2011). Effect of mixed flavonoids, *n*-3 fatty acids, and vitamin C on oxidative stress and antioxidant capacity before and after intense cycling. Int. J. Sport Nutr. Exerc. Metab..

[150] Davison G., Gleeson M., Phillips S. (2007). Antioxidant supplementation and immunoendocrine responses to prolonged exercise. Med. Sci. Sports Exerc..

